# Mussel-inspired dynamic facet-selective capping approach to highly uniform α-calcium sulfate hemihydrate crystals†

**DOI:** 10.1039/d3ra00835e

**Published:** 2023-05-22

**Authors:** Fu Feng, Yu-xin Cui, Yong-qi Hu, Sheng Hu, Ai-dong Zhang

**Affiliations:** a College of Chemistry and Environmental Engineering, Hubei Minzu University Enshi Hubei 445000 China fengfu2010@163.com; b Key Laboratory of Pesticide and Chemical Biology of Ministry of Education, College of Chemistry, Central China Normal University Wuhan 430079 China

## Abstract

We report herein a dynamic facet-selective capping (dFSC) strategy for α-calcium sulfate hemihydrate crystal growth from dihydrate gypsum in the presence of a catechol-derived PEI capping agent (DPA-PEI) with inspiration by the biomineralization process of mussel. The crystal shape is controllable and varies from long and pyramid-tipped prisms to thin hexagonal plate. The highly uniform truncated crystals have extremely high compression and bending strengths after hydration molding.

## Abstract 2

Shape evolution of α-calcium sulfate hemihydrate crystals with long prisms with top {111} facets to highly size- and shape-uniform thin hexagonal plates with solely top {002} facets.

## Graphical abstract

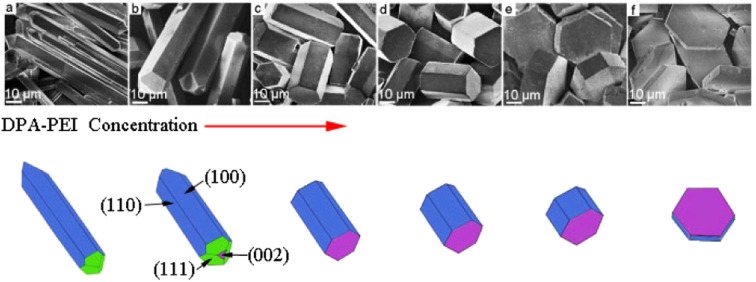

α-Calcium sulfate hemihydrate (α-HH) is an important cementitious material and is extensively used in the construction, ceramics and medical industries owing to its features of easy workability, fast setting time, high strength, and low cost.^1^ The applications are based on the hydration setting reaction of α-HH with optimum amounts of water leading to gypsum (dihydrate) crystallization and interlaced crystallites to form strong constructs. Since the hydration setting strengths are highly dependent on the morphology of α-HH crystals,^2^ controllable crystal growth of α-HH has attracted great attention because it provides a way to access interesting morphology dependent material properties and extensive applications. While in industry α-HH is produced mainly by the autoclaving process from dihydrate gypsum at elevated temperatures and pressures with high energy demand,^3^ the hydrothermal method, which operates under ambient pressure and relatively low temperatures with the presence of various additives, is an attractive alternative. This method may yield morphology-controlled α-HH crystals of high added-value, for example, for use as bone cement.^4^ To present, an array of factors, including solution pH, reaction temperature and additives have been explored. The use of additives is of paramount importance, and the additives, such as coexisting salts,^5^ small organic molecules,^6^ surfactants,^7^ even polymers,^8^ have been used to generate α-HH crystals in the shapes of short prisms, long columns, long whiskers, *etc.* Despite these efforts, however, to obtain excellent α-HH crystals with highly uniform size and desired aspect ratio *via* a controllable growth mechanism remains still a great challenge.^9–11^

The role of additives is to alter the growth habit of α-HH crystals, and based on the results from the α-HH crystal models,^12^ macroscopic spectroscopic analysis and molecular simulations,^13^ it was inferred that the additives selectively adsorb on side or top facets of crystal seeds and prevent further crystal growth in the direction, leading to differently shaped crystals. The inhibition of crystal growth along certain facets by the selective adsorption of biogenic species and bio-inspired additives is a common strategy adopted in natural and artificial mineralization to control the crystal morphology.^14^ Many bio-inspired materials have been reported to mimic the biological conditions for controllable crystal growth and mineralization, for instance, the hydroxyapatite rod-like nano-particles with high aspect ratios were synthesized through a biomimetic pathway using polyvinylpyrroliddone as the additive,^15^ while the uniform hexagonal vaterite CaCO_3_ mesocrystals were obtained with the biomimetic mineralization conditions using a cellulose derivative as the additive.^16^ To our knowledge, there is no report on the morphology-controllable synthesis of α-HH crystals with biomimetic conditions so far based on the transformation of calcium sulfate dihydrate.

Here we developed a dynamic facet-selective capping (dFSC) strategy to control the growth of α-HH crystals in aqueous suspension of gypsum under the hydrothermal conditions with the addition of a specific capping agent, namely 3-(3,4-dihydroxyphenyl) propionic acid grafted poly(ethylenimine) (DPA-PEI) (Scheme 1). This additive simultaneously contains the catechol units and poly(ethylenimine) backbone. The choice of DPA-PEI was inspired by the binding ability of the catechol unit with a number of metal ions and with virtually any solid surface.^17^ The catechol unit is of biogenic origin as one key component in mussel foot adhesive proteins.^18^ By taking use of the metal ion binding of mussel-inspired catechol-containing dopamine and polydopamine, the controlled crystal growth of unstable calcium carbonate vaterite microspheres^19^ and the anchoring mineralization of hydroxyapatite crystals on different surfaces^20^ have been demonstrated. Moreover, one analogue of DPA-PEI with a 60% acylation degree has been reported to have the ability to form substrate independent layer-by-layer assembly on virtually any surface by exploiting the strong interfacial binding property of this catechol containing polymer.^21^ On the other hand, poly(ethylenimine) is a basic substance that can be protonated in neutral and acidic conditions, which may provide additional function in crystal growth control. Thus the purpose of using DPA-PEI in the present work is twofold: selectively binding to Ca^2+^ rich facets of α-HH crystals and preventing the approach of Ca^2+^ in solution from exchanging the bound catechol moieties on crystal surface, the latter would be important to stabilize the predicted metastable {002} face of α-HH crystals.^22^ On the basis of this expectation, it is possible to obtain novel α-HH crystal morphologies by taking use of this dual role of the DPA-PEI polymer. To our knowledge, there is no report on shape-controlled growth of α-HH crystals under the presence of an additive containing catechol or poly(ethylenimine). Thus one DPA-PEI polymer, which possesses an acylation degree of 30% based on the primary amine content of PEI (branched PEI with a molecular weight of 20 kDa and a ratio of 1 : 2 : 1 for the primary, secondary and tertiary amine groups), was used for the morphology-controlled growth of α-HH crystals. The present work reports that, as expected, the highly uniform α-HH crystals with different shapes and aspect ratios can be facilely obtained under the presence of this DPA-PEI. With the high quality α-HH crystals in hand, a mechanism, oriented aggregation growth of α-HH crystals, was revealed based on the results from SEM and TEM characterizations, and morphology dependent strength after hydration setting were explored.

**Scheme 1:**
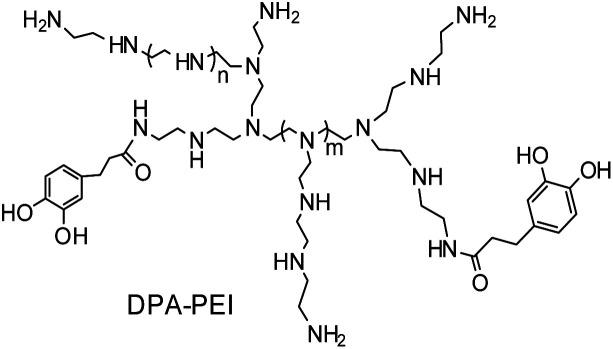
Schematic representation of the molecular structure of DPA-PEI as an additive for the controllable α-HH crystal growth.

The molecular structure of this DPA-PEI is shown in Scheme 1 and its synthesis, ^1^H NMR, FTIR spectra and GPC analysis results can be found in the ESI (Comment S2, Fig. S1, S2, and Table S1†). In the present work, we initiated a systematic investigation on the effect of this capping agent on the crystal growth by examining the size and shape of the resultant crystals, grown in the hydrothermal conditions and in the presence of DPA-PEI at varying concentrations of 0, 1.0, 2.0, 3.0, 4.0 and 5.0 g L^−1^, respectively, at pH 5.5 and evaluated temperature 97 ± 0.5 °C for 1.5 ∼ 7.0 h (Comment S3 in the ESI†). Fig. 1 shows the SEM images of these α-HH crystals. All these crystals have been confirmed to be pure α-HH phase using XRD and TG-DSC (Fig. S3 and S4 in the ESI†). Without using DPA-PEI, the α-HH crystals are not well uniform hexagonal prisms with average 100 μm in length and 10 μm in width, and the tops are terminated with several polygonal faces assigned as the {111} facets (Fig. 1a). When DPA-PEI was used at 1.0 g L^−1^, the crystals became uniform hexagonal prisms with average 80 μm in length and 15 μm in width, and each top is terminated with three tetragonal-like faces assigned as the {111} facets and one newly generated trigonal face as the {002} facets^23^ (Fig. 1b). When a higher concentration of DPA-PEI (2.0 g L^−1^) was employed, the crystals were tuned to be hexagonal prisms with average 50 μm in length and 20 μm in width, and each top is terminated with solely one hexagonal face assigned as the {002} facets, and thus the crystal is an octahedron (Fig. 1c). With further increase of the concentration of DPA-PEI to 3.0 g L^−1^, the crystals became further uniform hexagonal prisms with average 35 μm in length and 23.5 μm in width, and each top is also terminated with the {002} facets (Fig. 1d). When DPA-PEI was used at 4.0 g L^−1^, the crystals were tuned to be uniform hexagonal prisms with average 17.5 μm in length and 34.0 μm in width, and each top is also terminated with the {002} facets (Fig. 1e). With further increase of the concentration of DPA-PEI to 5.0 g L^−1^, the crystals were modulated to be perfectly uniform thin hexagonal plates with average 4 μm in length and 45 μm in width, and each top is terminated with the {002} facets, giving large-scale excellent uniformity of α-HH octahedron crystals (Fig. 1f and S5†).

**Fig. 1:**
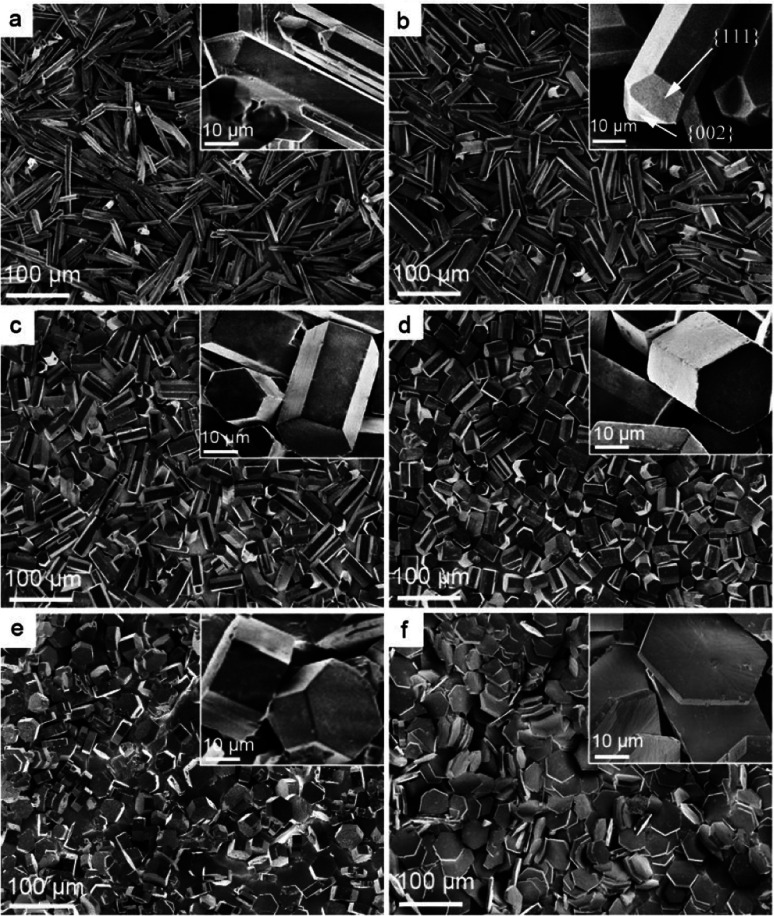
SEM patterns of α-HH crystals obtained at different DPA-PEI concentrations of (a) 0 g L^−1^, (b) 1.0 g L^−1^, (c) 2.0 g L^−1^, (d) 3.0 g L^−1^, (e) 4.0 g L^−1^, (f) 5.0 g L^−1^, and shape evolution of α-HH crystals: (a) long prisms with top {111} facets; (b) shortened prisms with top {111} facets and newly appeared {002} facets; (c) further shortened prisms topped with solely {002} facets; (d, e) highly size- and shape-uniform shortened prisms with solely top {002} facets; and (f) thin hexagonal plates with solely top {002} facets.

Due to the remarkable variations in size and shape of the α-HH crystals as directly seen in SEM characterization (Fig. 1), statistical analysis of the uniformity or monodispersity of the crystals in diameter and aspect ratio would give a quantitative sense with interest. To quantify the uniformity of these crystal particles, 40 particles of each sample were randomly selected to measure their diameters and aspect ratios. The data are depicted with color dots as shown in Fig. 2, and the regions of the main dot distribution in each sample are highlighted by respective annotated colors, representing intuitively the ranges of the crystal distribution observed in the α-HH crystal samples. The detailed statistical distributions in diameter and aspect ratio for the crystals are given in Fig. S6 and S7 (ESI†). It is clearly seen that the DAP-PEI concentration has a paramount effect on the uniformity of α-HH crystals in the growth process. The aspect ratios of these α-HH crystals obtained with the DPA-PEI concentrations at the range from 0 to 5.0 g L^−1^ were found to be 10.0, 5.3, 2.5, 1.5, 0.5 and 0.1 in average, respectively, suggesting a strong DPA-PEI concentration-dependence. Moreover, the aspect ratio of 0.1 for α-HH crystals grown at the DPA-PEI concentration of 5.0 g L^−1^ is the lowest one ever reported as far as we know.

**Fig. 2:**
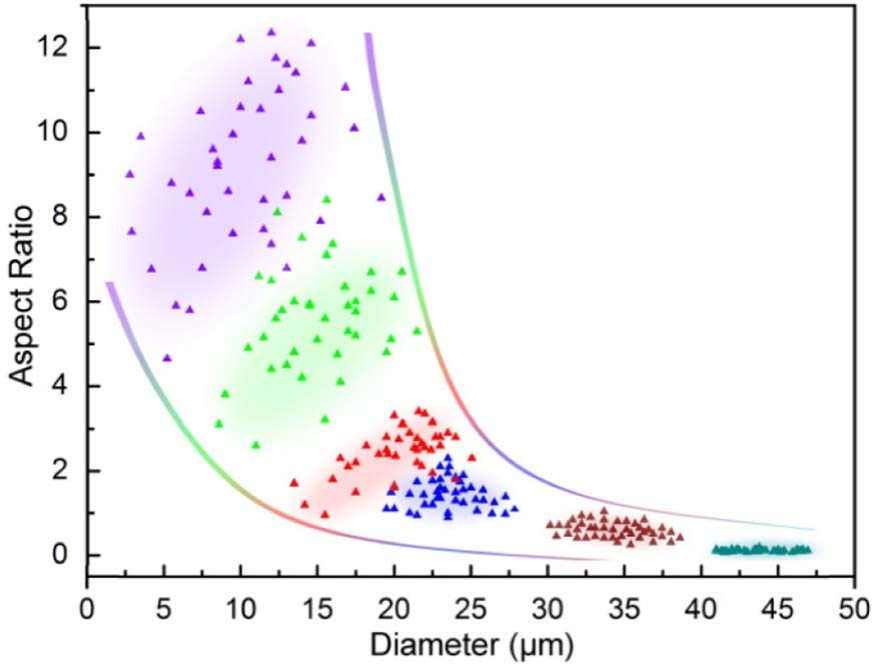
Distribution regions in diameter (*X*-axis) and aspect ratio (*Y*-axis) for 40 α-HH crystals randomly selected from the respective SEM mages (violet, green, red, blue, wine and dark cyan dots stand for the α-HH crystals grown in presence of DPA-PEI at the concentrations of 0, 1.0, 2.0, 3.0, 4.0 and 5.0 g L^−1^; the distribution areas of the predominant dots in each sample are colored to guide the eye).

The effect of pH was also examined on the α-HH crystal growth at a fixed concentration of DPA-PEI (2.0 g L^−1^) by using the solution pH higher or lower than 5.5. The SEM images of the crystals grown at pH 8.5, 7.0 and 4.0 are shown in Fig. S8 (ESI†). Compared to the morphology of α-HH crystals grown at pH 5.5 (Fig. 1c), the α-HH crystals yielded at pH 8.5 are long yet irregular hexagonal prisms with a larger average aspect ratio of ∼8.5. With decreasing the pH to 7.0, the crystals became a shape with aspect ratio and width similar to that obtained in the conditions of DPA-PEI at 1.0 g L^−1^ and pH at 5.5 (Fig. 1b). At pH 4.0, the crystals tended to form shapes similar to that grown at pH 8.5. The results suggest pH 5.5 is the optimum one for the α-HH crystal growth control.

These data collected in controlled crystal growth experiments confirm that the diameter and aspect ratio as well as the disappearance of {111} facet and the appearance of {002} facet of α-HH crystals are dominantly dependent on the concentration of DPA-PEI, though the solution pH also has a definitely effect. Furthermore, it is worthy of mention that the crystal growth was retarded with the presence of DPA-PEI. For the complete crystal transformation of gypsum to α-HH crystals, the reaction times from 1.5 h to 7.0 h were required with the increase of the DPA-PEI concentration from 0.0 to 5.0 g L^−1^ (Fig. S9 in the ESI†), leading to the respective crystals as observed in Fig. 1.

To measure the elemental compositions on different crystal facets, the energy-dispersive spectrometry (EDS) analysis was performed, and the results are shown in Fig. 3 in which the atomic abundances are labeled at the respective peaks. It is evidenced that the atom abundances on the side {100} and top {002} facets are significantly different. On the {100} facet, the crystal compositional elements Ca, S, and O have abundances of 13.79, 17.24, and 68.95%; whereas the {002} facet has corresponding abundances of 17.97, 14.98, and 61.39%, respectively, meaning more S on {100} and more Ca on {002}. The result is in accordance in atomic ratio with the elemental density calculations on these facets based on the molecular model of α-HH in the literature.^24^

**Fig. 3:**
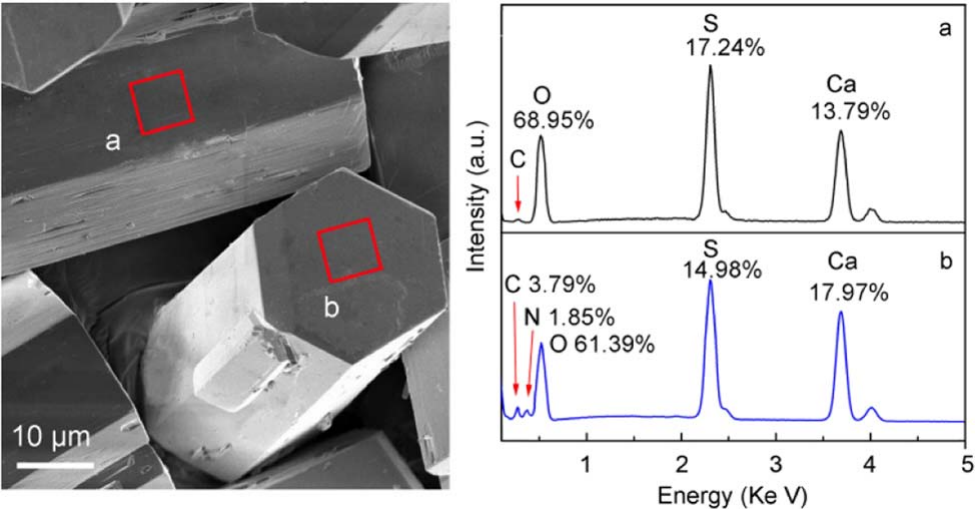
α-HH crystals showing the areas on the {100} (a) and {002} (b) facets for selective area EDS measurement and the elemental composition and respective abundances.

The adsorption of the additive DPA-PEI on α-HH crystal facets is the key to understand its role in the crystal growth. The FT-IR spectra of α-HH crystals verified the presence of DPA-PEI because of the appearance of the characteristic absorption bands of CH_2_ and NH groups at 2932/2850 and 3445 cm^−1^, respectively, and which gradually increase in relative intensity with the increase of employed DPA-PEI amount (Fig. S10 in the ESI†). Most importantly, the large enough {002} facets enabled us to perform the selective area EDS measurements on the crystal top surface for the first time and make a comparison with the crystal side facets in elemental composition. As shown in Fig. 3, only trace of element C was found on the {100} facets, whereas on the {002} facets, 3.79% of C and 1.85% of N elements were detected. This differentiation implies the distinct adsorption abilities of DPA-PEI on the {100} and {002} facets. The DPA-PEI molecule consists of catechol and ethylenimine units in which the catechol unit is able to bind with the cationic Ca element, thereby the more Ca^2+^ abundant {002} facet surely binds more DPA-PEI molecules than the more SO_4_^2−^ abundant {100} side facets.

The controllable crystal growth and the resultant novel morphologies of α-HH crystals manifest the specific role of the additive DPA-PEI, and based on the data obtained, it is possible to outline a plausible mechanism for the crystal growth process in the presence of DPA-PEI (Fig. 4). Under the hydrothermal conditions, dihydrate gypsum dissolves partially to give a supersaturated CaSO_4_ solution, and subsequent nucleation produces crystal seeds that transform to small rods within 1.0 h of growth as seen in the TEM image (Fig. S11†). The distribution differentiation of Ca^2+^ and SO_4_^2−^ on the {002} and {100}/{110} facets results, and in the presence of DPA-PEI, the catechol moieties carrying PEI unit bind more to the {002} facets than to the {100}/{110} facets, due to the catechol–Ca^2+^ interaction. Then two pathways are possible. In pathway A, under a low concentration of DPA-PEI (1.0 g L^−1^) and the optimum conditions of pH and salt concentration, low amount of DPA-PEI is adsorbed on the {002} facets, decelerating the growth along the *c*-axial of the crystal and leading to the tetradecahedron crystals topped with three {111} facets and one small {002} facets (Fig. 1b). In pathway B, at a higher concentration of DPA-PEI (3.0 g L^−1^, for instance), higher amount of DPA-PEI is adsorbed on the {002} facets, further decelerating the growth along the *c*-axis of the crystals, leading to the perfectly uniform octahedral prisms with tops of solely {002} facets (Fig. 1d).

**Fig. 4:**
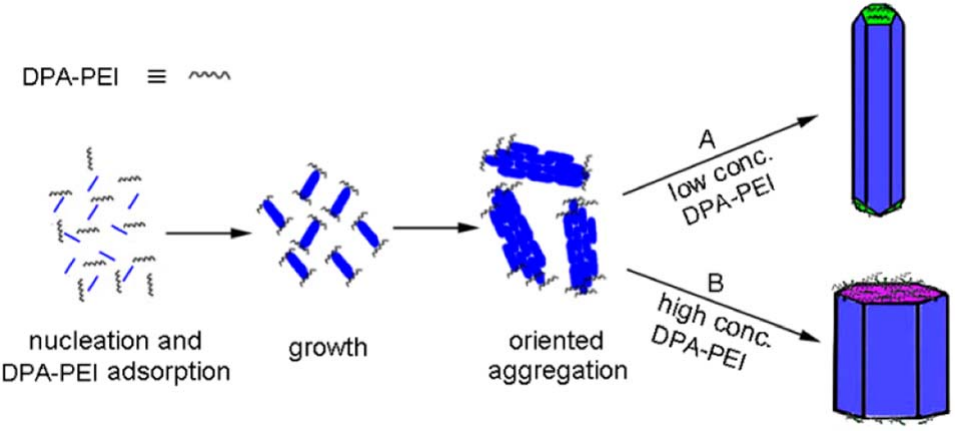
Schematic representation of the α-HH crystal growth processes at different concentrations of DPA-PEI.

At pH 5.5, DPA-PEI is likely protonated to a low extent on the ethyleneimine units while the catechol hydroxyl groups remains intact due to its high p*K*_1_ as reported to be 9.34.^25^ Thus the binding of catechol unit with surface presenting Ca^2+^ occurs likely *via* the two hydroxyl groups instead of by the binding of phenoxide anions. On the other hand, the slightly protonated PEI units may act the role as a barrier to prevent Ca^2+^ from penetrating the network of the adsorbed DPA-PEI layer and thereby to slow down the growth along the *c*-axial direction.

The selective adsorption of DPA-PEI on {002} facet should be a dynamic or equilibrium process to make the oriented aggregation growth possible, because the oriented aggregation growth needs interface fusing of nanorods to become larger crystals like the interface elimination and recrystallization occurring in the absence of true solution.^26^ In our case, the oriented aggregation state of nanorods in the initial growth stage is also firmly evidenced with TEM observation (Fig. S11†). Moreover, morphology evolution of the top {002} and side {100/010} faces with growth time revealed with SEM (Fig. S12 and S13, ESI†) confirmed the process of interface elimination and recrystallization. Therefore, weak and dynamic binding of DPA-PEI to crystal facets must be true. Previous reports have demonstrated the reversible binding of catechol to Fe^3+^, and to Ca^2+^ ions.^27^ This labile binding may stem from the reason that Ca^2+^ can not form strong coordinate bonds with catechol, rendering the dynamic binding possible in the hydrothermal conditions. Several times of washing with hot water can remove all the attached DPA-PEI as revealed by FT-IR.

The motivation to develop effective approaches to well shape-regular α-HH crystals comes from their interesting morphology-dependent fundamental material properties and the extensive applications. The mechanical strength after hydration setting is one of the primary considered parameters, which has proven to be related to the regularity, aspect ratio, size, and size distribution of α-HH crystals. The mechanical strengths of α-HH crystals after hydration setting were tested and the results are depicted in Fig. 5, showing the relations of compressive strength and flexural strength as a function of aspect ratio of α-HH crystals. It is clearly seen that with the decrease of aspect ratio, which was resulted from the addition of DPA-PEI at successively increased concentrations, the compressive and flexural strengths of the hydration setting are steadily increasing. The highest compressive and flexural strengths were measured to be 73.6 MPa and 31.2 MPa, respectively, when the aspect ratio is decreased down to about 0.1 as shown in Fig. 5 and 1f. The extremely high strength is presumably originated the lowest aspect ratio ever reported to date. In the gypsum industry, one custom is employed to grade α-HH crystals according to their hydration setting strengths as being α10, α20, α30, α40, and α50, with the latter to have the compressive and flexural strengths larger than 50 and 6 Mpa, respectively, as defined in the standard JC/T 2038-2010 (a High Strength Gypsum Plaster, China Construction Materials Industry Standards). The strengths are important reference indexes for choice in different applications. To our knowledge, the highest strengths of α-HH crystals in the present work are the best ones as compared to the reported strengths of α-HH crystals synthesized using the hydrothermal method at ambient pressure^28^ or the autoclaving method. Therefore, the lowest aspect ratio of 0.1 and the regularity of α-HH crystals produced in this work are the important parameters responsible to the highest strengths.

**Fig. 5:**
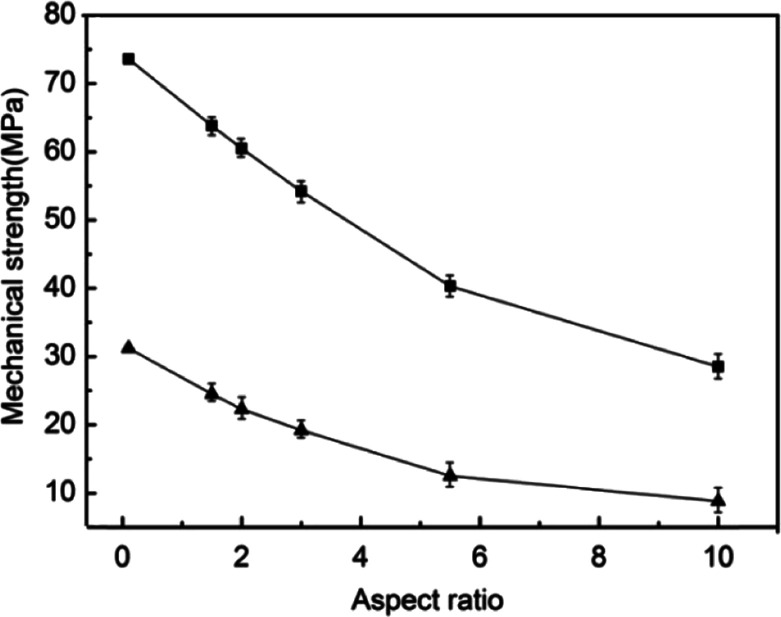
Relations of compressive strength (square) and flexural strength (triangle) as a function of aspect ratio of α-HH crystals after hydration setting.

## Conclusions

In this work, we developed a new capping agent for the crystal growth of α-HH by a dynamic facet-selective capping (dFSC) strategy, and the resultant crystals were shown to possess controllable size, aspect ratio, and regularity. The growth proceeded in a mechanism of oriented aggregation process controlled by the dynamic facet-selective binding of DPA-PEI on the crystal {002} facets. With the readily available large and regular crystals, the elemental composition and the adsorption of an additive on the {002} facets of α-HH crystals were measured using selected area EDS. The hydration setting exhibited the highest mechanic strengths, which is very important for various industrial applications. In addition, the strategy developed in this work is believed to be extendable to the controlled crystal growth of other inorganic materials.

## Conflicts of interest

There are no conflicts to declare.

## Supplementary Material

RA-013-D3RA00835E-s001
