# Supplementary material for: Mussel-inspired dynamic facet-selective capping approach to highly uniform α-calcium sulfate hemihydrate crystals
Source: RSC Adv. 2023 May 22;13(22):15342–6. doi: 10.1039/d3ra00835e (PMC10201340; doi:10.1039/d3ra00835e)
Supplement: RA-013-D3RA00835E-s001 [file RA-013-D3RA00835E-s001.pdf]

Electronic Supplementary Material (ESI) for RSC Advances.
This journal is © The Royal Society of Chemistry 2023

Supporting Information

Mussel-Inspired Dynamic Facet-Selective Capping Approach to Highly Uniform α-Calcium Sulfate Hemihydrate Crystals

Fu Feng[a], Yu-xin Cui[a], Yong-qi Hu[a], Sheng Hu[a] and Ai-dong Zhang*[b]

[a] College of Chemistry and Environmental Engineering, Hubei Minzu University, Enshi, Hubei 445000, China.

E-mail: fengfu2010@163.com

[b] Key Laboratory of Pesticide and Chemical Biology of Ministry of Education, College of Chemistry, Central China Normal University, Wuhan 430079, China.

Table of Contents

Comment S1 Materials and characterization for DPA-PEI and α-HH crystals...............................3
Comment S2 Syntheses of DPA-PEI...4
Comment S3 Controlled growth of α-HH crystals...5
Comment S4 Mechanical strength measurement...5

Fig. S1 ^{1}H NMR of DPA-PEI with a DPA substitution degree of 30%..6

Fig. S2 FT-IR spectrum of DPA-PEI with a DPA substitution degree of 30%...............................6

Fig. S3 XRD patterns of HH crystals synthesized at different concentrations…………..……....7

Fig. S4 TG-DSC curves of α-HH crystals synthesized at pH 5.5 and DPA-PEI 2. 0 g L^{-1}..............7

Fig. S5 SEM images of α-HH crystals with thin hexagonal plates..8

Fig. S6 Diameter distributions of α-HH crystals synthesized at different concentrations..............9

Fig. S7 Aspect ratio distributions of α-HH crystals synthesized at different concentrations..........10

Fig. S8 SEM images of α-HH crystals synthesized at different pH..10

Fig. S9 Concentration effect of DPA-PEI on the time for complete transformation of α-HH from gypsum (pH = 5.5)………..11

Fig. S10 FT-IR spectra of α-HH synthesized at different concentrations of DPA-PEI..................11

Fig. S11 TEM image of the oriented aggregation state of nanorods after growth for 1 h.............12

Fig. S12 {002}facets evolution of α-HH crystals revealed by SEM in full-view and high-resolution SEM images with different time...12

Fig. S13 (110)/(010) face evolution of α-HH crystals revealed by SEM in full-view and high-resolution SEM images with different time..13

Table S1 Data of Molecular weights of DPA-PEI with 30% DPA measured with GPC in triplicate..13

References...14

Comment S1: Materials and characterization for DPA-PEI and α-HH crystals

Materials: All of the chemicals and solvents used in the experiment were of analytical grade without further purification. Gypsum ($CaSO_4·2H_2O$), calcium chloride, potassium chloride, sodium hydroxide, and hydrochloric acid were purchased from Sinopharm Chemical Reagent Co., Ltd. Dialysis bags (with cut off molecular weight of 3 kDa) were purchased from Shanghai Green Bird Science and Technology Development Co. (Shanghai, China). 3-(3,4-Dihydroxyphenyl) propionic acid (DPA) (98%), branch poly(ethylenimine) (PEI) (MW = 20 kDa with a ratio of 1:2:1 of primary: secondary: tertiary amines), and N-ethyl-N'-(3-dimethylaminopropyl)-carbodimide hydrochloride (EDC) (99 %) were purchased from Sigma-Aldrich.

Characterization: Powder X-ray diffraction (XRD) patterns were recorded on a Rigaku Rotaflex Dmax-2500 X-ray diffractometer with Cu Kα radiation (λ = 1.5418 Å). Scanning electron microscopy (SEM) measurements were performed on a JEOL JSM-6700F microscope operated at 5 kV. Transmission electron microscopic (TEM) images were acquired on a JEOL JEM-2100F microscope with accelerating voltage of 200 kV. Thermogravimetry and differential scanning calorimetry (TG-DSC) were performed on a STA-409PC analyzer (Netzsh, Germany) with a heating rate of 5 °C min^{-1} in a nitrogen atmosphere. Fourier transform infrared (FT-IR) characterizations were done on a Bruker Tensor 27 FTIR spectrometer. ^1H NMR spectra were collected on a Varian Mercury Plus-400 MHz spectrometer using D_2O as the solvent and TMS as an internal standard. Compressive and flexural strengths of the crystals after hydration setting were performed on a MTS-810 analyzer (MTS, USA) following the procedure in accordance with ASTM C 472-1999, and the result was expressed as an average value of at least three independent determinations.

Comment S2: Synthesis of 3-(3,4-dihydroxyphenyl)propionic acid grafted poly(ethylenimine) (DPA-PEI)

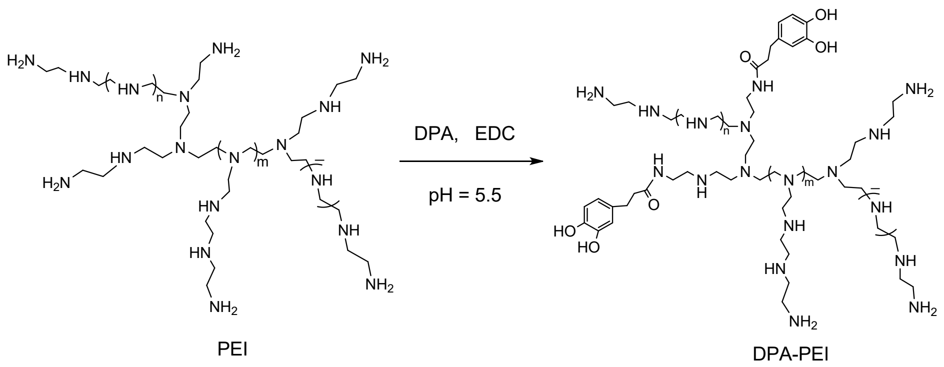

Synthetic route of DPA-PEI.

The synthesis was performed by following a reported procedure[1] with some modification, and exemplified using synthesis of the DPA-PEI with a 30% substation degree. Briefly, PEI (2.0 g, M_w = 20000 g mol^{-1}) was dissolved in 200 mL of deionized water adjusted to pH 5.5 using 1 M HCl solution. 3-(3,4-dihydroxyphenyl)propionic acid (DPA,1.06 g, 5.8 mmol) and EDC (2.24 g, 11.6 mmol) were added. The reaction was carried out at room temperature for 2.5 h during which the solution pH was kept at 5.5 by adding proper amount of 1.0 M NaOH. After reaction, the resultant mixture was dialyzed using a dialysis membrane against distilled water for 3 d to remove unreacted chemicals and byproducts. The substitution degree of DPA on the PEI chain was estimated to be 30% by the ninhydrin test.[2] The polymer has a weight-average molecular weight (M_w) of 26365 g mol^{-1} as characterized with GPC and a isoelectric point (pI) of 9.41 determined by a capillary isoelectric focusing-whole column imaging detection.[3] The molecular structure of the polymer was characterized by ^{1}H NMR and FT-IR.

^{1}H NMR (400 MHz, D_2O): δ = 2.42 (t, *J* = 8.0 Hz, 2H, Ar-CH_2), 2.40 (t, *J* = 8.0 Hz, 2H, O=C-CH_2), 2.69(m, 53H, PEI-CH_2), 6.55 (d, *J* = 8.0 Hz, 1H, Ar), 6.67(s, 1H, Ar), 6.72 (d, *J* = 8.0 Hz, 1H, Ar) ppm. FT-IR (KBr) *v*: 3445 s (NH), 2932-2850 s (CH_2), 1652 m (CO-NH); 1525 w (NH); 1475 w (Ar); 1043 s (C-N) cm^{-1}.

Comment S3: Controlled growth of α-HH crystals

A mixed solution (60 mL H_2O, 15.5 g $CaCl_2$, 0.5 g KCl and DPA-PEI, 0-5.0 g L^{-1}, Mw = 26365 g mol^{-1}) with different pH (4.0~8.5) was added into a 250 mL three-neck flask reactor equipped with a glass condenser. Then 47 g of gypsum ($CaSO_4 \cdot 2H_2O$) was introduced to form a slurry with stirring by a Teflon impeller at a constant rate of 180 rpm. The mixed solution was heated at a fixed temperature (97 ± 0.5 °C). During the reaction, aliquots of 5 mL slurry were withdrawn at fixed time intervals and filtrated by vacuum filtration. The solid samples were immediately washed with boiling deionized water twice and dried at 45 °C in an oven for 2 h for characterizations.

Comment S4: Mechanical strength measurement

Compressive and bending strengths of hydration molding of α-HH

The preparation of hydration molding of α-HH was done according to the procedures descried in GB/T 17669.3 (China national standard for gypsum plasters). The weight ratio of water to α-HH crystals in modeling was 0.32. The paste was cast into a stainless steel mold with the size of 20 × 20 × 80 mm and compacted by jolting. After maintained for 2 h, the molding block was demolded and stored in a curing chest maintained at 25 ± 2 °C and 65 ± 5% RH for 7-10 days. The molding block was tested for bending strength measurement with three-point loading in a span of 50 mm. The loading rate was 0.02 kN/s. The compressive strength was measured with a loading area of 0.0004 m^2 and loading rate of 0.6 kN/s. All measurements were done in triplicate.

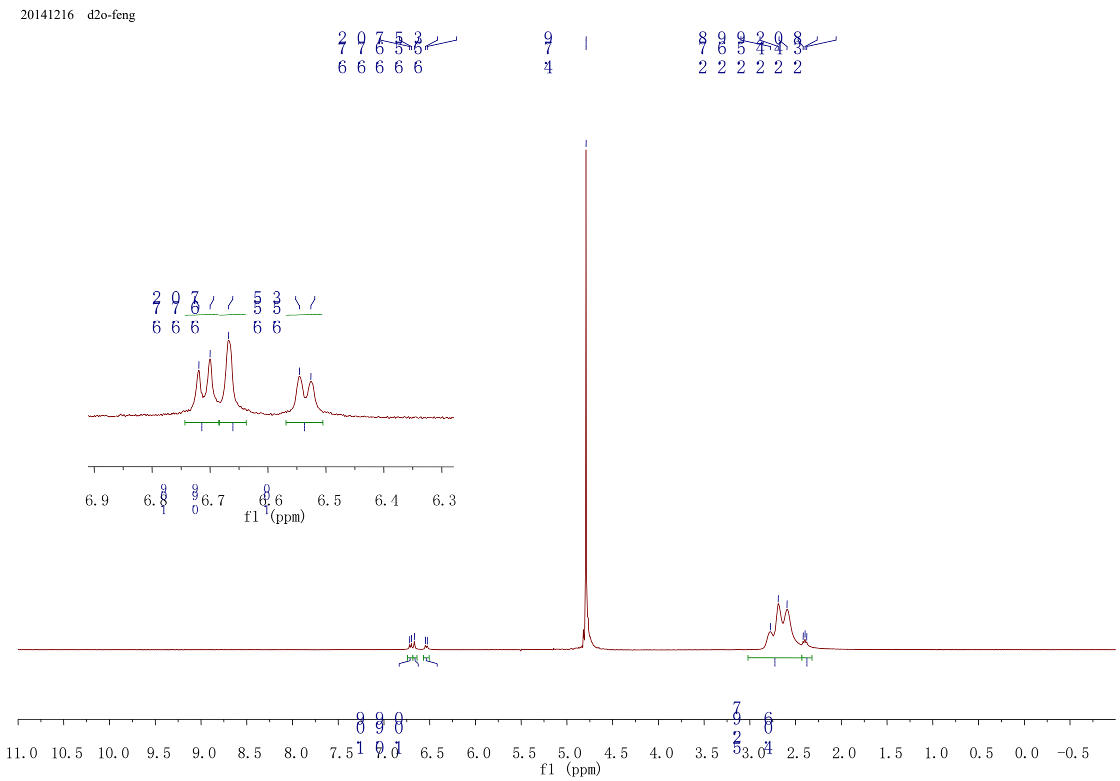

Fig. S1 ^{1}H NMR of DPA-PEI with a DPA substitution degree of 30%.

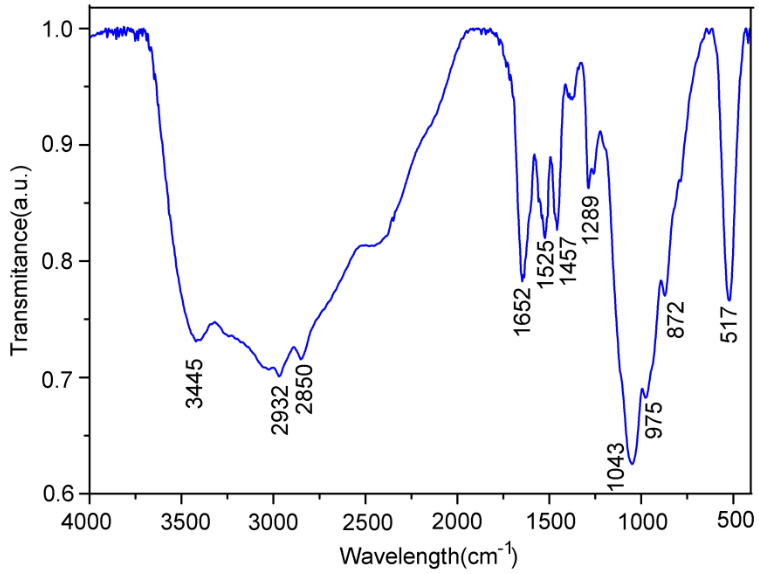

Fig. S2 FT-IR spectrum of DPA-PEI with a DPA substitution degree of 30%.

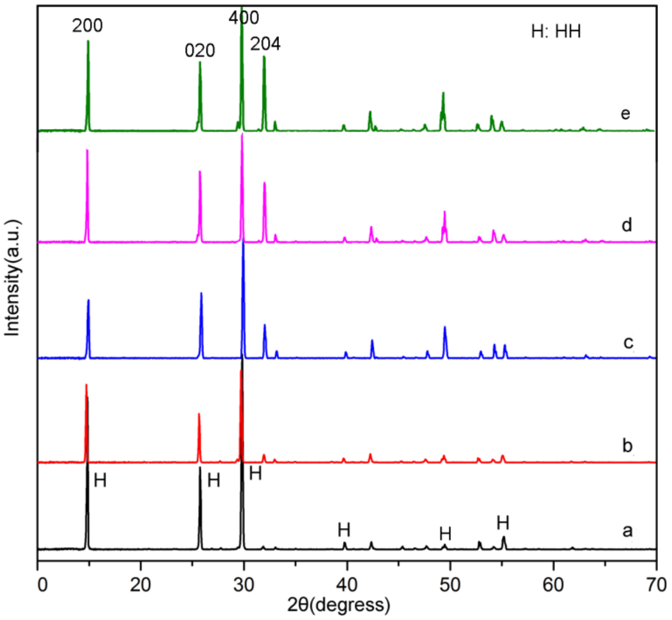

Fig. S3 XRD patterns of HH crystals synthesized at different concentrations.

(DPA-PEI: (a) 0.0 g L^{-1}, (b) 1.0 g L^{-1}, (c) 2.0 g L^{-1}, (d) 3.0 g L^{-1}, (e) 5.0 g L^{-1} (pH = 5.5)).

The XRD patterns show main peaks at 2θ of 14.7°, 25.6°, and 29.7°, which can be iundexed to a pure HH phase (PDF-ICDD 047-0964).

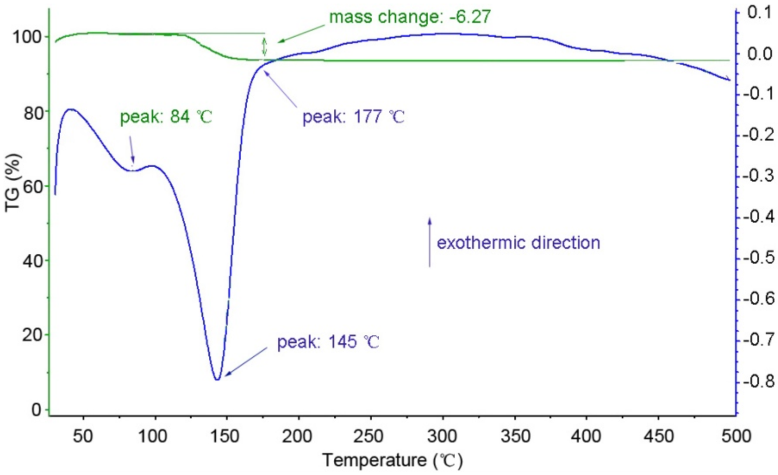

Fig. S4 TG-DSC curves of α-HH crystals synthesized at pH 5.5 and DPA-PEI 2. 0 g L^{-1}.

The TG curve reveals a water release of 6.27 wt % when the crystals are heated from 132 to 180 ℃, which is approximate to the theoretical crystal water content of 6.21 % in α-HH. The simultaneous DSC curve shows an endothermic peak at ~145 °C, followed by an exothermic peak

at ~177 ℃, which is the characteristic profile of the α-HH crystal phase.[4] Thus the obtained crystals are pure α-HH crystals, in accordance with the result from XRD.

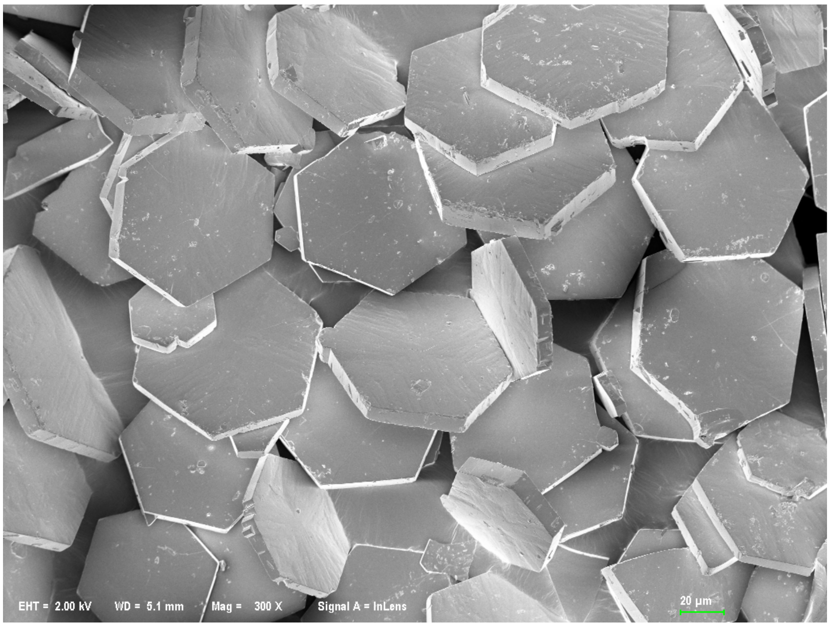

Fig. S5 SEM images of α-HH crystals with thin hexagonal plates.

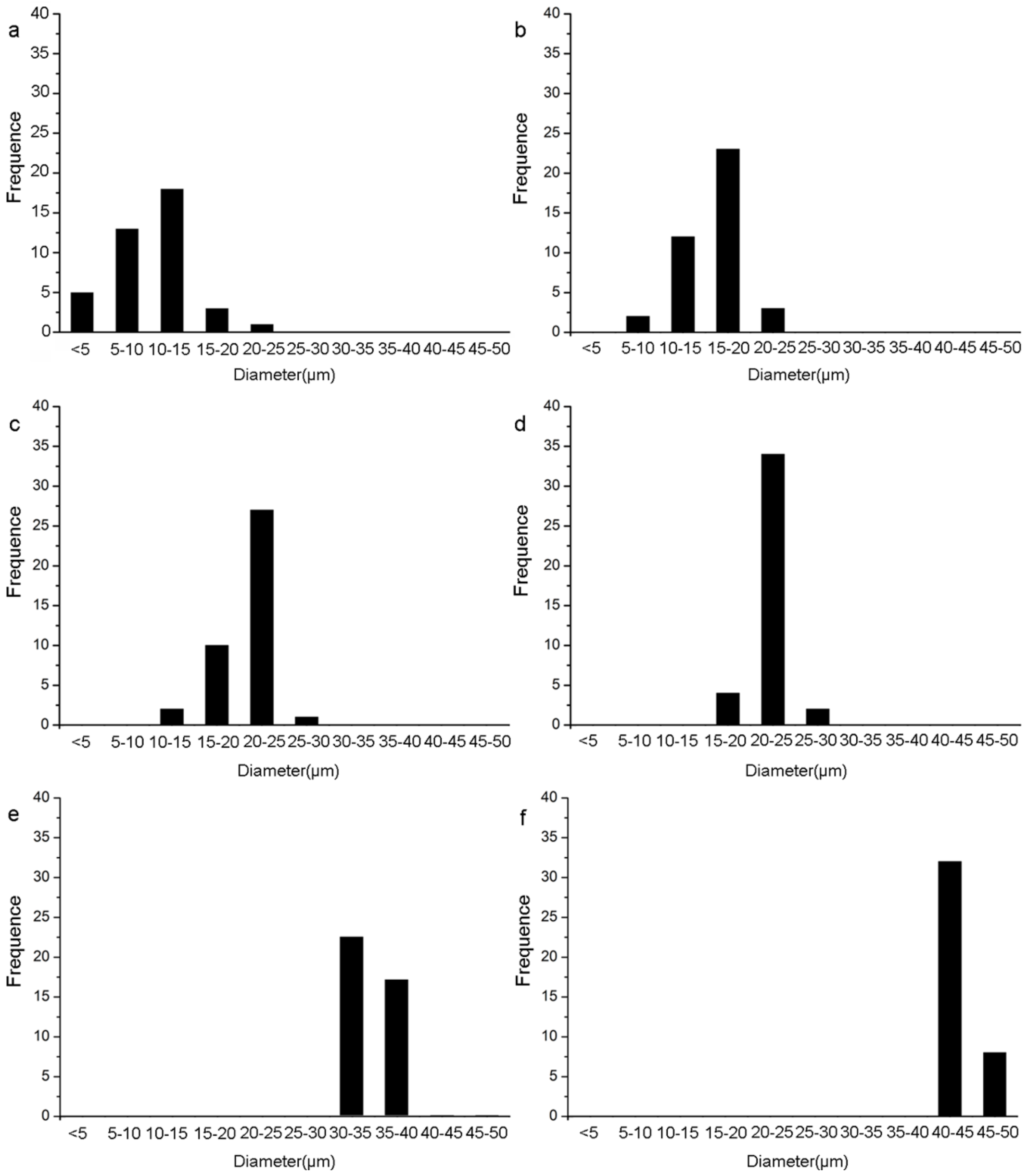

Fig. S6 Diameter distributions of α-HH crystals synthesized at different concentrations. (DPA-PEI: (a) 0.0 g L^{-1}, (b) 1.0 g L^{-1}, (c) 2.0 g L^{-1}, (d) 3.0 g L^{-1}, (e) 4.0 g L^{-1}, (f) 5.0 g L^{-1} (pH = 5.5)).

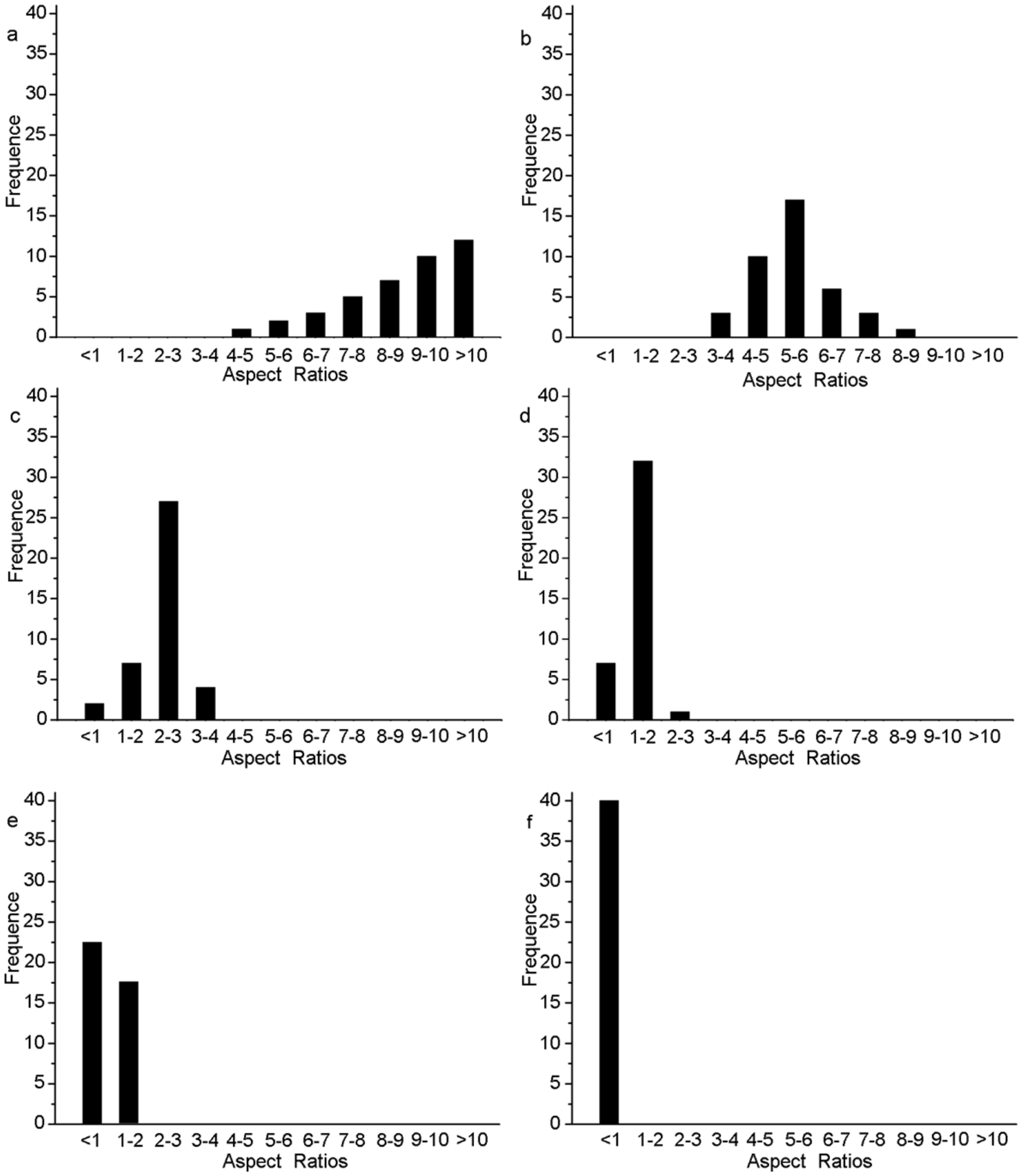

Fig. S7 Aspect ratio distributions of α-HH crystals synthesized at different concentrations.

(DPA-PEI: (a) 0.0 g L^{-1}, (b) 1.0 g L^{-1}, (c) 2.0 g L^{-1}, (d) 3.0 g L^{-1}, (e) 4.0 g L^{-1}, (f) 5.0 g L^{-1}

(pH = 5.5)).

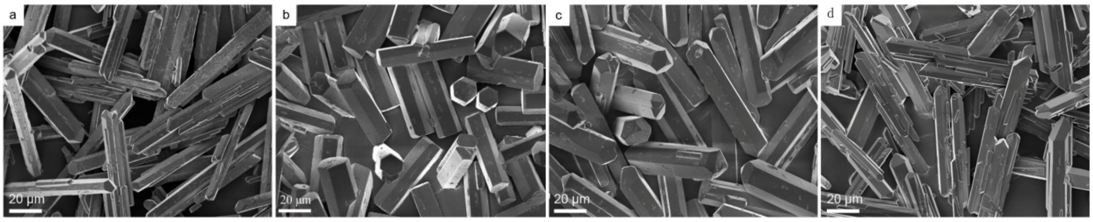

Fig. S8 SEM images of α-HH crystals synthesized at different pH.

((a) pH 4.0, (b) pH 5.5 ,(c) pH 7.0, (d) pH 8.5 (DPA-PEI = 2. 0 g L^{-1})).

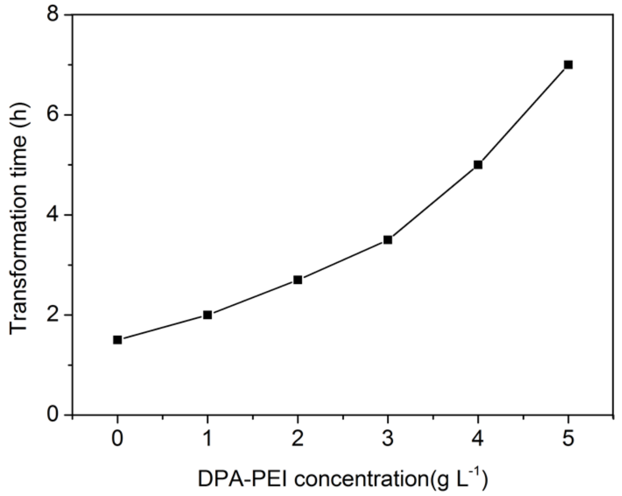

Fig. S9 Concentration effect of DPA-PEI on the time for complete transformation of α-HH from gypsum (pH = 5.5).

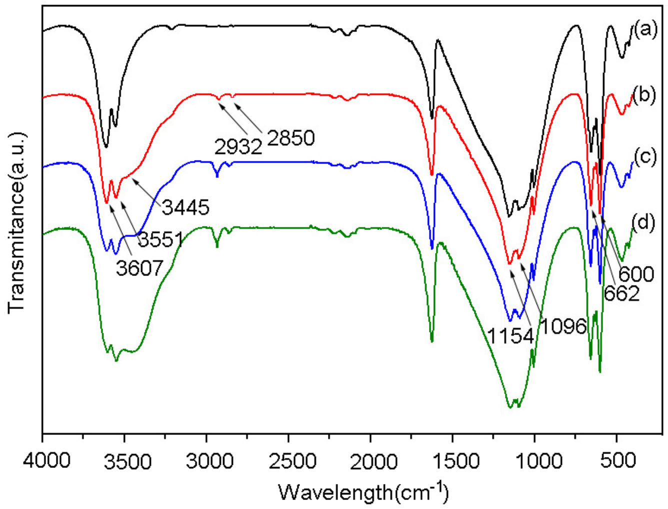

Fig. S10 FT-IR spectra of α-HH synthesized at different concentrations of DPA-PEI.

((a) 0.0 g L^{-1}, (b) 1.0 g L^{-1}, (c) 3.0 g L^{-1}, (d) 5.0 g L^{-1} (pH = 5.5)).

Fig. S10 shows the FT-IR spectra of α-HH synthesized without and with the existence of DPA-PEI. The bands at 1154, 1096, 662 and 600 cm^{-1} can be assigned to SO_4^{2-} stretches and the bands at 3607 and 3551 cm^{-1} can be assigned to O-H stretching. The bands at 3445 cm^{-1} can be assigned to N-H stretching, which increases in width with an increase in the concentration of DPA-PEI (Figure S9b-d). Two bands are observed in the region of 2800-3000 cm^{-1}, which are assigned to the asymmetric (2932 cm^{-1}) and symmetric (2850 cm^{-1}) stretching vibrations of CH_2. The data indicate the presence of DPA-PEI on crystal surface.

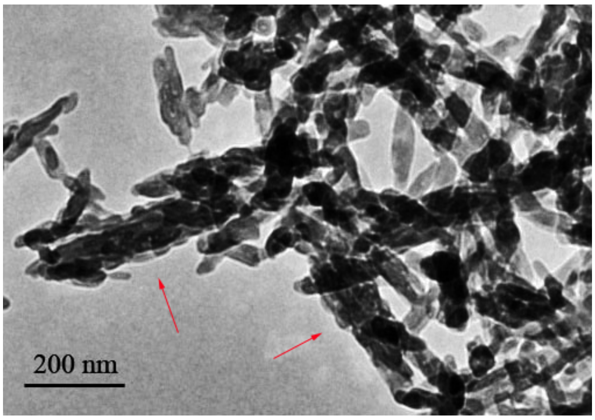

Figure S11. TEM image of the oriented aggregation state of nanorods after growth for 1 h. (at DPA-PEI 1.0 g L^{-1} and pH 5.5. Red arrows highlight the co-oriented nanorods in the aggregates).

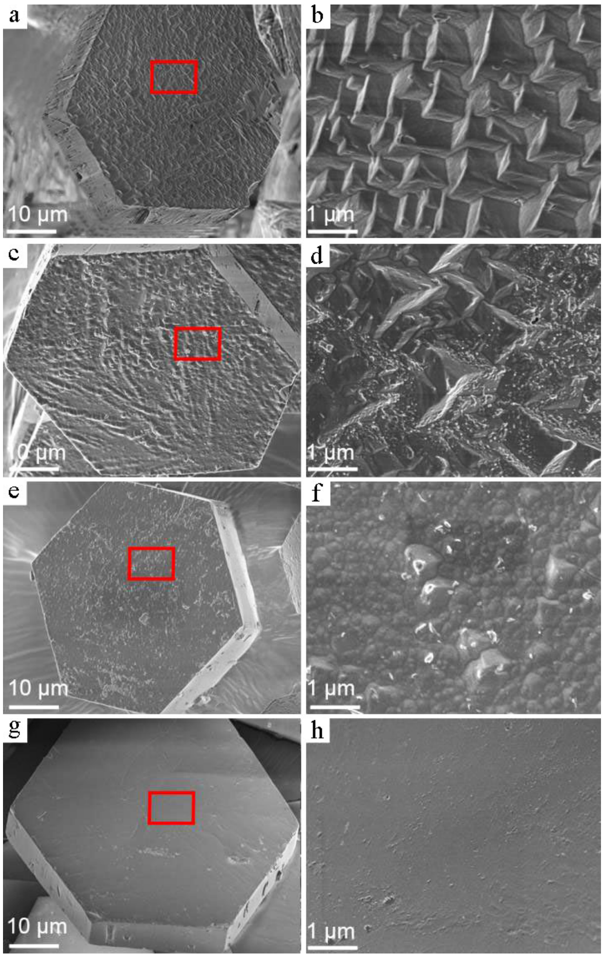

Fig. S12 {002} facets evolution of α-HH crystals revealed by SEM in full-view and high-resolution SEM images with different time.

((a,b) 4.0 h; (c,d) 4.5 h; (e,f) 5.5 h; (g,h) 7.0 h, $C_{DPA\text{-}PEI}$ = 5.0 g L^{-1}, pH = 5.5).

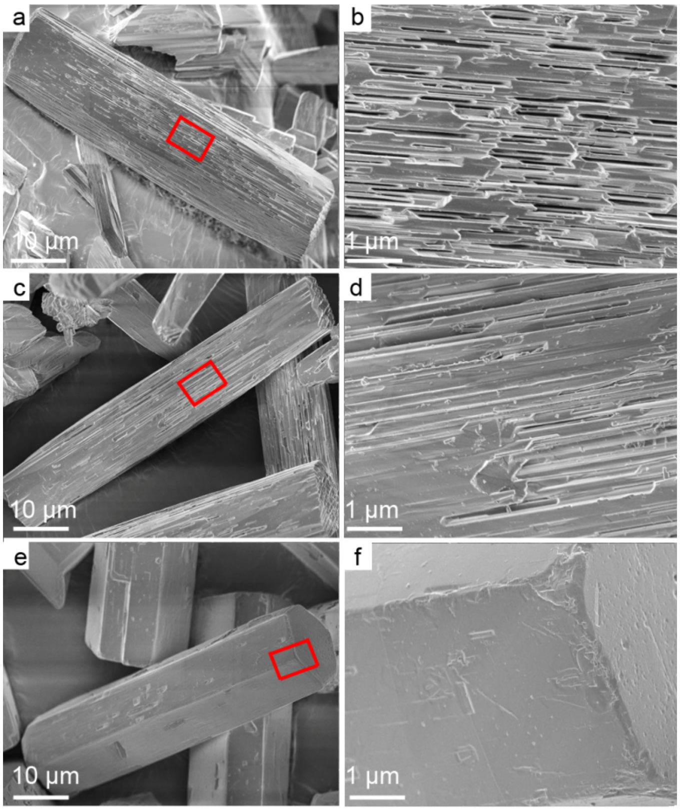

Fig. S13 (110)/(010) face evolution of α-HH crystals revealed by SEM in full-view and high-resolution SEM images with different time.

((a,b) 2.0 h; (c,d) 2.5 h; (e,f) 3.5 h, $C_{DPA\text{-}PEI} = 2.0$ g L^{-1}, pH = 5.5).

Table S1. Data of Molecular weights of DPA-PEI with 30% DPA measured with GPC in triplicate.

test 1	test 2	test 3	average
$M_n = 12063.39$	$M_n = 8686.82$	$M_n = 9507.49$	$M_n = 10085.9$
$M_w = 26177.56$	$M_w = 26581.68$	$M_w = 26335.76$	$M_w = 26365.00$
$M_w/M_n = 2.17$	$M_w/M_n = 3.06$	$M_w/M_n = 2.77$	$M_w/M_n = 2.61$

References

(1) Lee, H. S.; Lee, Y. H.; Statz, A. R.; Rho, J. S.; Park, T. G. Messersmith, P. B. *Adv. Mater.* **2008**, *20*, 1619-1623.

(2) Prochazkova, S.; Vårum, K. M.; Østgaard, K. *Carbohydr. Polym*. **1999**, *38*, 115-122.

(3) Goodridge, L.; Goodridge, C.; Wu, J.; Griffiths, M.; Pawliszyn, J. *Anal. Chem.* **2004**, *76*, 48-52.

(4) Guan, B. H.; Yang, L. C.; Wu, Z. B.; Shen, Z. X. Ma, X. F. Ye, Q. Q. *Fuel* **2009**, *88*,1286-1293.
